# Global localization of 3D point clouds in building outline maps of urban outdoor environments

**DOI:** 10.1007/s41315-017-0038-2

**Published:** 2017-11-22

**Authors:** Christian Landsiedel, Dirk Wollherr

**Affiliations:** Chair of Automatic Control Engineering, Technische Universität München, Munich, Germany

**Keywords:** Global localization, Semantic mapping, Hybrid mapping, Point clouds

## Abstract

This paper presents a method to localize a robot in a global coordinate frame based on a sparse 2D map containing outlines of building and road network information and no location prior information. Its input is a single 3D laser scan of the surroundings of the robot. The approach extends the generic *chamfer matching* template matching technique from image processing by including visibility analysis in the cost function. Thus, the observed building planes are matched to the expected view of the corresponding map section instead of to the entire map, which makes a more accurate matching possible. Since this formulation operates on generic edge maps from visual sensors, the matching formulation can be expected to generalize to other input data, e.g., from monocular or stereo cameras. The method is evaluated on two large datasets collected in different real-world urban settings and compared to a baseline method from literature and to the standard chamfer matching approach, where it shows considerable performance benefits, as well as the feasibility of global localization based on sparse building outline data.

## Introduction

Accurate localization in urban environments is a crucial dependency of many emerging robotic applications, such as autonomous vehicles, delivery and service robots, or augmented reality applications. While systems like the global navigation satellite system (GNSS) or localization based on wireless signals are sufficient for many applications, there is a benefit to a robot being able to localize based purely on its own sensors in cases these external services are unavailable or lacking in accuracy. In urban and highly structured environments, large, usually artificial, planar structures provide robust features for localization and registration of 3D sensor data (Pathak et al. 2010). Many vertical planes in urban environments are represented in human-readable maps as building outlines, such that a mapping between the two allows to localize a robot in the global map coordinate frame. This paper describes a method to perform this localization based on data from a 3D laser range finder, for example for a robot travelling in an urban environment, in a 2D map containing building outlines. Such map information is freely available from common online map sources like OpenStreetMap (Haklay and Weber 2008), Google Street Maps or official municipal cadastral maps. The proposed localization method uses only information about building outlines and the street network, which keeps its demands for storage capacity or bandwidth low. It is based on the geometry of the environment alone, without the requirement of visual features such as appearance or texture data. Thus, it is largely independent from seasonal variation or variation based on the time of day. The matching procedure needs a single 3D laser scan as input. Therefore, no odometry or time series of measurements is necessary. As a global localization method using an external map, it is not necessary for the robot to have visited the location before or to build a feature database for the purpose of localization, since all necessary map information is freely available online.

The localization problem as posed here is an instance of the template matching problem: finding a relation between the query features, consisting of the planar segments in the robot observation, and the building outlines in the map. Theoretically, this problem could be solved by knowing the correspondence between a single observed plane and one building edge in the map; however, this correspondence problem is highly nontrivial, especially when no appearance information is used.

As for all localization methods, the environment needs to contain a sufficient amount of salient information to uniquely distinguish it; the lack of this uniqueness is known as *perceptual aliasing*. For a localization method that builds on geometry alone, this means it will not perform well in very highly structured or highly artificial environments, but our experiments show that there is sufficient information contained for the method to work for a large part of two different real-life urban environments containing scenes with varying urban characteristics such as streets with high building density, tunnels, courtyards and open spaces.

This paper is structured as follows: in Sect. 2, the proposed method is categorized with respect to the different localization tasks important in robotics, and an overview over related work is given. Section 3 describes in detail the steps performed to estimate the robot pose in the building outline map. The approach is experimentally evaluated on two datasets and compared to a baseline method in Sect. 4. Section 5 concludes the paper.

## Related work

Localization is a field of research that, due to its crucial importance for the successful operation of autonomous robots, has received extensive attention from the scientific community. For a categorization of the different methods and approaches discussed in this overview over related work, it is helpful to distinguish a number of related robotics problems associated with localization.

- *Place recognition* is the problem of matching sensor data collected in a place to a database of features collected in a number of distinct places, and retrieving the correct one. To build this database, the robot has to have visited all eligible places before.
- *Simultaneous localization and mapping (SLAM)* describes the process of building a consistent metric map of an environment, which then can be used for localization. The input usually consists of a sequence of distance measurements from a laser scanner or similar sensor and odometry information, while other sensor measurements, for example about appearance, can be incorporated as well. An initial pose estimate, e.g., the result of a global localization method, is needed for starting the SLAM process.
- *Semantic localization* is sometimes used for the process of labeling the surroundings of the robot based on sensor data (image or otherwise) with semantic categories (Vasudevan and Siegwart 2008; Rubio et al. 2016; Drouilly et al. 2014). Even though this is not a problem of localizing a robot on a map, its result can be used as part of such a localization method as an additional feature.
- *Global localization* or the *kidnapped robot problem*, which is the topic of this work, describes the task of localizing a robot on a map in a global frame without any prior information. For general applicability, it is desirable that the map comes from an external source, such as a topographical or cadastral map, and does not have to be built based on sensor measurements specifically for the purpose of localization. Usually, global localization should work from a single sensor measurement or a short sequence of measurements, such that it can be used as initialization procedure, for example for SLAM as described above.

Further distinctions between localization methods for robots in urban environments can be made based on the sensors that are used to provide observations about the environment. Many robots are equipped with a GPS sensor, which often provides information about the global location of the robot, which however may be noisy or temporarily unavailable due to obstructions in the environment. Other methods are based on camera images, either from monocular cameras or images with attached depth information from stereo cameras. Laser distance measurements and odometry measurements are often used as inputs in SLAM localization methods, while *visual SLAM* relies on monocular or stereo camera images.

The following gives an overview over different recent attempts at localization in urban areas, moving from appearance-based methods to ones that use semantic features of the environment. Finally, the approaches that localize on maps of building outlines, such as the one presented in this work, are surveyed.

For global localization approaches, different kinds of maps have been considered as a reference against which to determine the location of the robot. Many approaches have focused on using appearance data for localization. Common to these is a databases of sensory images annotated with location information, against which a query image is matched to retrieve the camera location. The following paragraph gives an overview over these approaches.

Aerial images have been used as prior information for localization in approaches such as the one by Leung et al. (2008), which extracts line segments from street-level images and matches the geometric relationships derived from them to aerial orthoimagery using a particle filter. Another example for this group of methods is the work by Kümmerle et al. (2011), which presented a SLAM system that uses aerial images as a global prior. It matches structures found in aerial images to laser data, and uses the relationships as constraints in graph-based SLAM. Agarwal et al. (2015) showed how to improve an approximate location estimate by matching short series of camera views with Google Street View panoramic images. This method enables global localization in an area of about 1 km radius. Majdik et al. (2013) presented a similar approach for the localization of flying vehicles in the Google Street View image database, where the difference in viewpoint between the images taken from the street level and the images from flying height constitutes a challenge. Localization in indoor environments modeled by a database of 2.5D images was shown by Liang et al. (2015). Their approach divides the localization problem into a place recognition step, where a template image is retrieved from the database, and a subsequent pose matching between the query and the template image. Cappelle et al. (2007) compared robot observations with images sampled from a highly accurate dataset of 3D geometry and RGB appearance data to determine the robot position in cases where for example GPS is not available. A database of street-level image data augmented with 3D building models was used in the work of Baatz et al. (2012) to localize a device just from monocular images, where the geometry of the query image is approximated with vanishing point detection.

A second group of approaches does not rely on appearance data, but uses sparser maps containing different sets of semantic features of urban environments for localization. For moving robots with the capability of estimating their trajectory, this knowledge can be used to localize the robot by comparing the travelled path with the paths that are feasible in the road network. Lee et al. (2007) integrated approximate digital maps of the road network as additional constraints with a SLAM framework based on traditional on-board sensors. The OpenStreetSLAM system (Floros et al. 2013) uses chamfer matching to compare a trajectory of a robot, which is determined with visual odometry, to street map information. It localizes the robot by tracking pose hypotheses in a particle filter and selecting those which fit best with the paths traversable on the road network. Gupta and Yilmaz (2016) and Brubaker et al. (2013) followed similar approaches, but used different representations for the travelled trajectories, which allowed for different matching formalisms. Irie et al. (2016) presented a localization mechanism on high-level street maps containing street as well as sidewalk outlines that relies on labeling streets in images and retrieving a matching map position using a dependence maximisation approach. The method put forward by Ruchti et al. (2015) also depends on the labeling of areas as street or non-street in laser scans. The semantic labeling results are used as sensor measurements in Monte Carlo localization on a map containing the street network of an urban environment. In a different approach presented by Hentschel and Wagner (2010), buildings extracted from OpenStreetMap were used as the reference map in a Monte Carlo localization framework. Vysotska and Stachniss (2016) used building outlines retrieved from laser scan data to improve the localization in a SLAM framework. In that work, the matching of local surrounding buildings with a 2D map was performed using the ICP algorithm (Besl and McKay 1992), which was used to provide additional constraints for a graph-based SLAM formulation. In contrast to these approaches, the localization method presented here aims at global localization, where no sequence of observations and no odometry data are available.

Building outlines in urban environments provide a salient source of geometric information, which has also been used for pose estimation with a single frame of sensor data. Many of these approaches have been based on estimating the geometry of the surroundings of the robot from camera images, and then estimating the camera pose in the map by finding matches with elements from the map data. For example, Antigny et al. (2016) used distinctive objects with the same appearance and constant, known dimensions (billboards etc.) which are contained in semantically annotated maps, and localized with respect to them. This allows users to refine a rough position estimate, which is used to select the road furniture object, to an accurate pose. Cham et al. (2010) performed localization in a 2D map based on a single omnidirectional ground level image, where the geometry of buildings was estimated using line and vanishing point detection, and geometric hashing was used to look up the transformation of the camera pose with respect to the map frame. The work presented by Chu et al. (2014) builds on this approach, but uses a similar method to refine the position retrieved from a GPS device, i.e., localize in a smaller area around a given position. The method also relies on extracting building edges from a monocular camera image and matching the resulting geometry of a single building to buildings contained in a 2D map. Arth et al. (2015) used monocular images and an initial GPS fix to localize in a 2.5D map. The ground plane of the map contains the building outlines contained in OpenStreetMap, whereas building heights were manually annotated. Matching was done by extracting lines from the camera images and matching them to the 2.5D map; additional filtering was executed by performing a semantic segmentation of the image and matching this against OpenStreetMap information.

This paper is most closely related to the approach of Cham et al. (2010), but it works on data from a laser scanner instead of on omnidirectional images, and uses the fact that building outlines are made up from line segments, for which the chosen matching method of chamfer matching is suited well. Furthermore, the proposed method includes visibility analysis for a more accurate matching between expected and actual observations. It also relaxes the assumption that multiple corners of a building need to be visible at the same time, which can be difficult in urban scenarios with large buildings, and particularly with occlusions. While the results presented in that work show that it is possible to reduce the number of candidate poses with the method presented there, a reliable global localisation without additional information cannot be based on it alone. Similar differences exist between the present work and the approach of Chu et al. (2014), which furthermore has the different goal of refining the position estimate received from a GPS device, and not global localization. This is also a relevant difference between the work presented here and the approach of Arth et al. (2015), where again the localization problem is solved for the case where monocular images of a location are available along with a location estimate from GPS or a similar sensor. Arth et al. (2015) also performed a step of rescoring pose hypotheses by comparing the input data with the content of the map that is visible from the candidate location, which is related to the formulation of the cost function taking into account visibility information put forward in this article. However, their method relies on performing a semantic classification of an input camera image and comparing it with a backprojection of map data including building height, which is different from the information available in the scenario envisioned here. In this work, the input data is given by a 3D laser scans, and the matching is done against building outline data alone. Evaluation shows that the method performs well in a region significantly bigger than the typical error of a GPS device, such that the method can be said to perform global localization on an urban scale, rather than GPS pose refinement using additional sensor data.

## Description of the localization method

### Method overview

The global localization method described in this paper uses 3D laser scans as sensor input data. It is matched against a 2D map of an urban environment, which contains information about building outlines as well as the street network. Data of this type can be retrieved from various sources, such as Google Maps, official cadastral maps or the OpenStreetMap project, which is used for the evaluation in this article.

**Fig. 1:**
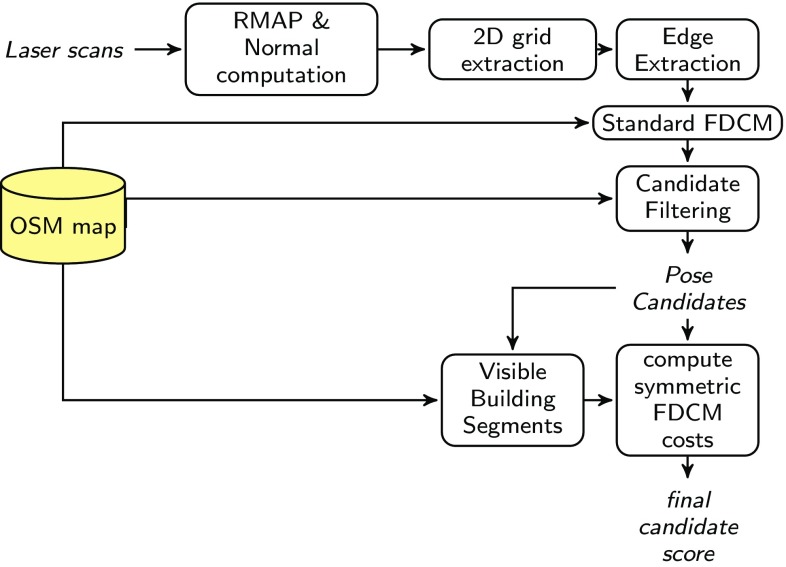
Sequence of operations performed for localization on the building outline map

The sensor data used for localization in this article comes from a 3D laser scanner. Only distance data is used, although appearance data in the form of laser intensities is often also available. Since the localization problem as discussed here is a 2D template matching problem, the initially 3D sensor data is reduced to a 2D representation by extracting vertical planar segments from the data, and reducing it further to a set of line segments representing these presumed building outlines. Matches between the building edges from the sensor data and the 2D building outline map are computed using a fast and simple template matching procedure known from image processing. Since the template matching problem for mapping has special properties which are not taken into account by standard procedures, the results of this approach can be improved upon. Information from the building map and street network are also used to further reduce the number of candidates valid for subsequent processing. The remaining candidate poses are then further refined by a variation of the chamfer matching procedure, which takes into account visibility considerations particular to the laser data matching problem, and penalizes matches where buildings that are absent in the sensor data appear in the corresponding map section. The result of this computation is used to rank the candidates and either extract the top candidate as the estimated pose, or use a ranked set of candidates for further processing, e.g., for the initialization of a SLAM system. The sequence of processing steps is also illustrated in Fig. 1.

### Point cloud processing and building outline segment detection

Before the template matching problem of localizing the robot on the building outline map can be addressed, the input data must be reduced to a set of lines representing the presumed building outlines in the sensor’s field of view. To this end, the very dense point clouds are reduced in size as a first step. For this, the rectangular cuboid approximation framework (RMAP) (Khan et al. 2014) is used to convert the point cloud into an occupancy grid consisting of cuboid cells at a lower resolution, and reduce the number of noisy observations. In this data structure, normal vectors can be efficiently computed for each occupied cuboid cell. Since we are interested in building outlines, and the roll and pitch angles of the robot travelling on the street can be assumed to be known, vertical surfaces can be extracted from the occupancy grid by selecting cuboid cells that have a normal vector parallel to the ground plane.

**Fig. 2:**
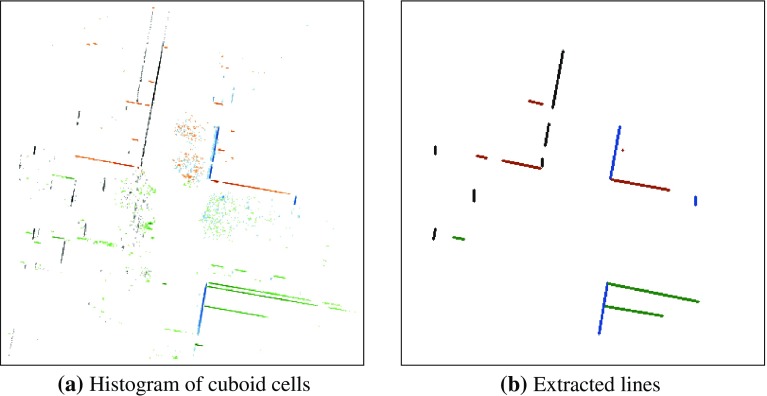
Illustration of the line segment extraction based on normal direction. The elements in the plots are colored according to the orientation of their normal vector

These vertically oriented cuboid cells are then projected to the ground plane by setting their *z* coordinate to zero, and the number of cells per area unit is counted. The result is a histogram of the vertically oriented cuboid cells in the sensor range of the robot. For the goal of extracting building outlines from this representation, the normal information from the point cloud should be preserved, since only points that have a similar normal direction can belong to a common planar surface. We use this information by binning the yaw angles of the cuboid cells and creating separate histograms for each angle range. In each of these histograms, line segments are extracted using the Probabilistic Hough Transform (Matas et al. 2000). Parallel line segments with small distances between them and collinear lines with small gaps are merged to reduce noise in the resulting set of edges. The building outline extraction process is illustrated in Fig.  2, which shows both the histograms of oriented cuboids, and the line segments computed based on them.

### Adapting directional chamfer matching to the localization problem

After the building outlines have been retrieved from the laser data, retrieving the robot pose in the building map becomes a template matching problem. Chamfer matching (Barrow et al. 1977) is a well-established method for template matching, which is especially suitable to find correspondences between sets of line segments. This section describes the idea of chamfer matching and extensions of its original cost function to adapt it to the problem of matching templates for localization.

Chamfer matching is designed to find a transformation of a template edge map in the robot coordinate frame $U = \left\{{\mathbf{u}}_i\right\} , i = 1, \ldots , n$ such that it optimally matches a section of a query edge map $V = \left\{ {\mathbf{v}}_i\right\} , i = 1, \ldots , m$ in the map coordinate frame. This transformation is a 2D Euclidean transformation ${\mathbf{s}} \in SE(2)$, where ${\mathbf{s}} = (\theta , t_x, t_y)$. It can be interpreted to define a pose of the robot in the coordinate frame of the map, where its location is given by $(t_x, t_y)$, and its heading by $\theta$. The effect of this transformation on the robot measurements can be calculated by a rotation and a subsequent translation as

1 $$\begin{aligned} {\mathbf{W}}({\mathbf{x; \,s}}) = \left( \begin{matrix} \cos (\theta ) & - \sin (\theta )\\ \sin (\theta ) & \cos (\theta ) \end{matrix} \right) {\mathbf{x}} + \left( \begin{matrix} t_x \\ t_y \end{matrix}\right) \end{aligned}$$

The optimal alignment of the query edge map with the template map is the result of the transformation which minimizes a distance function *d* between the two maps

2 $$\begin{aligned} \hat{{\mathbf{s}}} = \mathop {\arg\!\min }\limits_{{{\mathbf{s}} \in SE(2)}} d\left( {{\mathbf{W}}(U,s),V} \right). \end{aligned}$$

In the following, let the transformed query edge set ${\mathbf{W}}(U, s)$ be denoted by $\hat{U}$.

Different distance functions can be used. For standard Chamfer matching, the distance function is given by the minimal distances to a template edge point for each point in the query edge map

3 $$\begin{aligned} d_{CM} \left( \hat{U}, V\right) = \frac{1}{n} \sum _{\hat{\mathbf{u}}_i \in \hat{U}} \min _{{\mathbf{v}}_j \in V} \left| \hat{\mathbf{u}}_i - {\mathbf{v}}_j \right| . \end{aligned}$$

For edge maps consisting of linear segments, it is more robust and efficient to consider the orientation for the edge, and penalize matches between edge points with different directions. This reasoning leads to the distance function of directional Chamfer Matching (DCM) (Liu et al. 2010)

4 $$\begin{aligned} d_{DCM} \left( \hat{U}, V\right) = \frac{1}{n} \sum _{\hat{\mathbf{u}}_i \in \hat{U}} \min _{{\mathbf{v}}_j \in V} \left| \hat{\mathbf{u}}_i - {\mathbf{v}}_j \right| + \lambda \left| \phi (\hat{\mathbf{u}}_i) - \phi ({\mathbf{v}}_j)\right| , \end{aligned}$$

where an edge orientation $\phi$ is determined for each edge point, and the distance of the orientations is determined as the minimal rotation necessary between them. In applications where it is acceptable to discretize the space of edge orientations, the optimization (2) can be efficiently computed by computing a distance transform tensor, which contains the cost contributions for each query edge point. This approximation is formulated in the *fast directional chamfer matching* (FDCM) method (Liu et al. 2010). In cases where the template and query edge maps can be represented as sets of linear segments, the summation of individual contributions per point can be replaced by computations only involving the end points of the line segments by computing an integral distance transform.

**Fig. 3:**
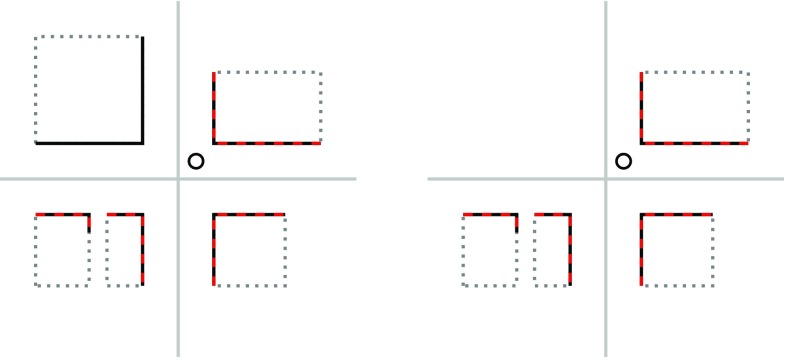
Illustration of examples for street corners with identical CM score, even though there are no sensor percepts of the building on the top left in the left-hand example. The candidate robot position is marked with a circle. Building outlines contained in the map are drawn dotted in grey, and their visible part in black. Lines extracted from a laser scan a drawn dashed in red. The street center lines are drawn in grey

These cost functions are designed for the task of finding simple query edge maps in template edge maps derived from cluttered images. It is expected that, for a good match between template and query edge map transformation, each edge in the query edge map is close to a matching edge in the template edge map. All edges at larger distances are not considered for cost computation. For the application of localizing a set of building edges in a building outline map, where, due to the structured nature of typical building maps, there can be many areas that are similar to parts of what the robot sensors observe, it is desirable to also penalize matches where some part of the template that should exist in the query is not there. This is illustrated in Fig. 3, which shows two possible transformations of a template edge map, both of which result in the same (D)CM cost values, but one of them is clearly a worse match than the other, since the building edges derived from the scan do not contain a building that would be expected to be observed.

While this information about which edges of the template map should be matched to edges in the query map is not available in a general template matching task, an estimate of the expected observation for the localization task can be generated by extracting all the lines visible in the map from a given robot pose. We denote this set of edges visible from a position $(t_x, t_y)$ by $V_e(t_x, t_y)$. With this definition, a *forward* cost function that takes only the expected observations for a given robot position into account can be defined as

5 $$\begin{aligned} d_{f}\left( \hat{U}, V, {\mathbf{s}}\right) = \frac{1}{n} \sum _{\hat{{\mathbf{u}}}_i} \min _{{\mathbf{v}}_j \in V_e(t_x, t_y)} \left| \hat{{\mathbf{u}}}_i - {\mathbf{v}}_{j}\right| . \end{aligned}$$

Furthermore, knowledge about the expected observation also allows to define a *reverse* cost function that describes the extent to which the expected observation $V_e$ is represented in the actual observation $\hat{U}$

6 $$\begin{aligned} d_{r}\left( \hat{U}, V, {\mathbf{s}}\right) = \frac{1}{n} \sum _{{\mathbf{v}}_i \in V_e(t_x, t_y)} \min _{{\mathbf{u}}_j \in \hat{U}} \left| \hat{\mathbf{u}}_i - {\mathbf{v}}_{j}\right| . \end{aligned}$$

Finally, the forward cost (5) and reverse cost (6) can be combined to form a cost function that is *symmetric* in the expected template edge map and the query map

7 $$\begin{aligned} d_{s}\left( \hat{U}, V, {\mathbf{s}}\right) = \frac{1}{2} \left( d_{f}\left( \hat{U}, V, {\mathbf{s}}\right) + d_{r}\left( \hat{U}, V, {\mathbf{s}}\right) \right) . \end{aligned}$$

A directional extension of these latter three cost functions similar to (4) is possible analogously.

Computing the optimization (2) for these latter cost functions is significantly more complex than the cost functions (3) and (4), as the set of visible edges, which constitute the template edge map used in the computation of the cost function, depends on the translation of the considered coordinate frame transformation. This means that a computation of a distance transform tensor, which is independent of the coordinate transformation and allows the efficient computation in the FDCM approach, is not possible when the area covered by the template map is large. Even though visibility analysis can be implemented efficiently using a Binary Space Partition (BSP) tree (Fuchs et al. 1983), a brute force optimization of (2) with either cost function $d_f$, $d_r$, or $d_s$ can be prohibitively computationally expensive. For this reason, in this work we adopt a heuristic approach by assuming that minimizers of these cost functions also result in low values of the simpler cost function $d_{DCM}$, if not the globally optimal ones. Under this assumption, the FDCM method can be used in a first pass to generate a set of pose candidates $C = \{{\mathbf{c}}_i\} = \{(\theta _i, t_{x, i}, t_{y, i})\}, i=1,\ldots , n_C$ that result in values of $d_{DCM}$ within a given factor of its global minimum. Only for these transformations, the visible lines are computed, and the more complex cost functions are evaluated.

### Filtering position candidates using OpenStreetMap information

**Fig. 4:**
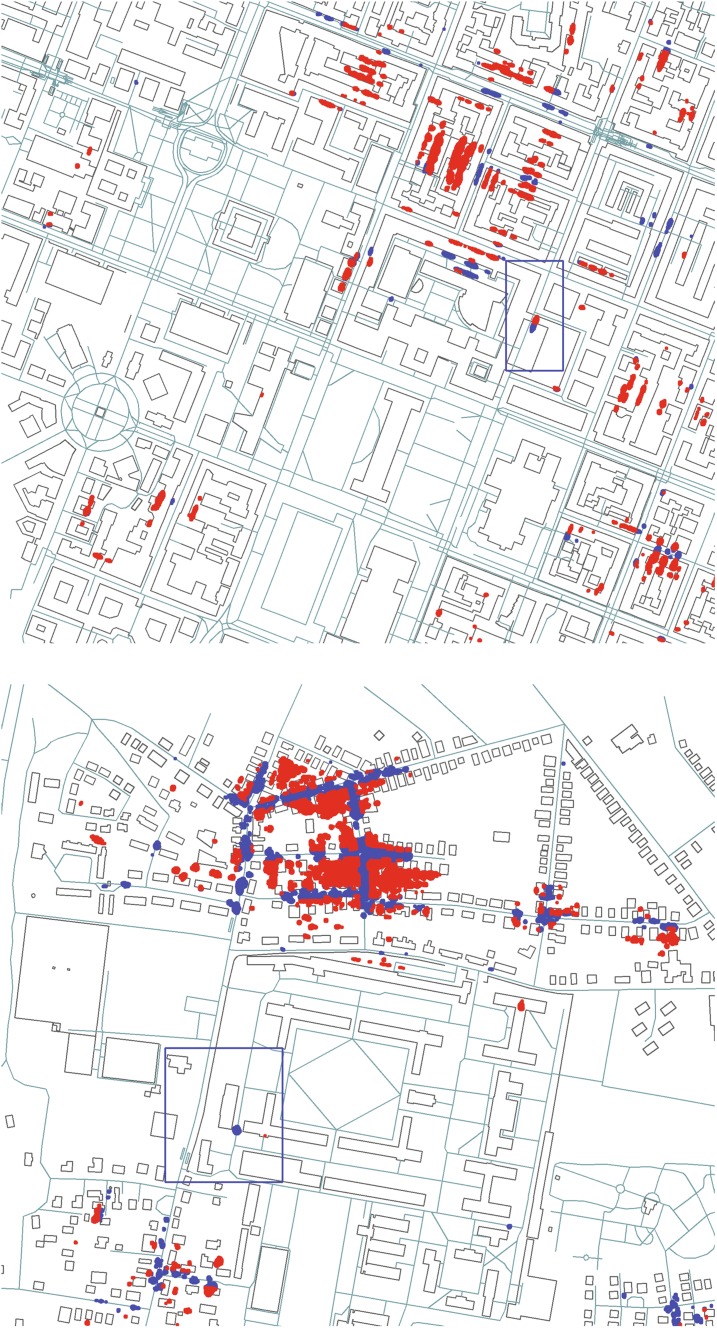
Filtering results for one scan with a large number of pose candidates from the Munich (top) and Bremen (bottom) datasets, respectively. Possible robot positions are marked with dots in red for invalid and blue for valid locations. The actual area covered by the corresponding scan is marked with a blue frame

The number of poses to consider for valid localization candidates can be restricted further with additional knowledge available from the building map. For instance, poses that lie inside buildings can be discarded. Furthermore, if, like in our case, the robot travels alongside the road, poses that are more than a given distance removed from any edge of the road network can be discarded as well. For the experiments carried out in this paper, we consider only pose candidates that are less than 12 m removed from street elements in the OpenStreetMap network. Of the many candidate poses generated by the first FDCM optimization, many are invalid according to either their position inside a building or their distance from a marked road, and thus do not have to be considered for further evaluation. This is illustrated in Fig. 4, which shows the positions of candidate poses for an input scene that produces many matches within the area considered for localization. The figure visualizes which points are considered as valid candidates and which ones are discarded based on the criteria laid out above.

## Experiments

The global localization method described above was evaluated extensively for localization accuracy. Data from two different datasets of urban environments with different characteristics were used for the evaluation. A baseline approach from literature was implemented for comparison, and the benefit of using the extended cost functions described in Sect. 3.3 over the standard DCM approach is shown.

### Dataset

The global localization method described in Sect. 3 was evaluated on 3D laser scans from two datasets. The Munich Urban Dataset (Wollherr et al. 2016) contains 80 scans covering an area around the inner city campus of the Technical University of Munich. It was recorded with a Zoller and Fröhlich 5010C 3D laser range finder and also contains RGB data. Scans were manually registered by annotating salient points in overlapping scans and finding the transformation that minimizes the error between the transformed positions of these scans. The Jacobs University Bremen dataset^1^ covers the campus of that university with 132 scans and was recorded with a Riegl VZ-400 laser scanner. The scans in this dataset were registered using reflective markers. Both datasets were manually aligned with the data retrieved from OpenStreetMap in a global coordinate frame.

### Experimental setup

As template data for the localization experiments, map data from OpenStreetMap was downloaded for a rectangular area of about 2 km width around the area covered by each dataset. This was used to generate the template building outline edge maps. The implementation of FDCM from Liu et al. (2010) was used to obtain the candidate poses with quantization of line orientations to 12 different direction channels. The grid size for the discretization of the positions that are searched by FDCM was set to 0.5 m. All poses that yielded a cost within a factor of 1.6 of the globally optimal FDCM cost were considered as candidate poses for further processing. The three cost functions newly proposed in Sect. 3.3 as well as the original FDCM cost were used to compute a final ranking of the pose candidates.

To the best of the authors’ knowledge, the only method from literature that has the same goal of global localization on building outline data alone and can thus serve as a baseline is the template matching method based on geometric hashing from Cham et al. (2010). Later methods that are based on this (Chu et al. 2014; Arth et al. 2015) use a similar matching method, but with added information in form of a GPS estimate, which is not available for the purpose of global localization. For a comparison with these prior methods, we implemented a hashing-based method similar to the one used in Cham et al. (2010) to be used with scale-invariant laser data, and measurements from the urban environments represented in the experimental datasets. It relies on extracting building corners from the building outline map, which are indexed with a hash function encoding building side length and the angle between the two sides belonging to the corner.

To localize a scan using the baseline method, first, the same edge extraction process as described for the proposed method is applied. Then, corners are found in the extracted line segments, and all matching corners from the map are retrieved using the hash index. The transformation between the corner and the laser scanner position is computed and applied to all matching corners from the map. The resulting poses are recorded in an accumulator, such that poses where multiple corners in the map are observed from the same scanner pose receive a higher score. From this accumulator, the cells with the highest scores of matching positions are retrieved as final scanner pose estimates. The parameters for quantizing the accumulator were optimized in a coarse grid search on the experimental data to a cell size of 5 m and a pose quantization that distinguishes six different orientations. Localization candidates were determined for each scan in both datasets using this baseline method.

### Experimental results

For the evaluation of the proposed localization methods, we focus on the error in the pose of the lowest-cost pose candidates. For this analysis, a pose candidate for which both the displacement as well as the rotational error with respect to the ground truth pose are below a threshold is denoted as *accurate*. For the proposed methods, these thresholds were chosen as 4 m and 0.2 radians as maximum displacement and rotational error, respectively. For the hashing-based method, the thresholds for determining whether a candidate is accurate were chosen to reflect the size of the grid used for the accumulator, which results in a maximal distance of 5 m and an allowed rotation of $\pi /6$. Note that this pair of thresholds is less strict than the one used for the proposed method. These numbers were chosen to allow for some error in the ground truth registration with respect to the OpenStreetMap map data, and to be significantly smaller than the typical error of GPS localization in urban areas (Zandbergen and Barbeau 2011).

For each dataset, the evaluation is performed in the number of scans $N_{\mathrm {accurate}}$ for which the set of *k* candidates with the lowest cost within a circular area of radius *w* around the ground truth position contains a candidate with an accurate pose. Thus, for the strictest evaluation criterion $k = 1$, a match means that the candidate pose with the lowest cost is accurate with respect to the given thresholds; for $k = 5$ it means that there is at least one accurate candidate among the 5 candidates with least costs.

The proposed method with the chosen parametrization produced a set of pose candidates containing an accurate pose candidate for 73 of the 80 scans in the Munich dataset, and for 119 of the 132 scans in the Bremen dataset. The average number of candidates per scan for the full map used for the experiments before filtering was 1845, and 472 after filtering based on street and building data for the Munich dataset; for the Bremen dataset these numbers were 13,254 and 5581, respectively. Forward and symmetric costs were computed for a maximum of 500 pose candidates with the lowest FDCM costs per scan because of their high computational demands with the current implementation; all other pose candidates were not evaluated for these costs.

**Fig. 5:**
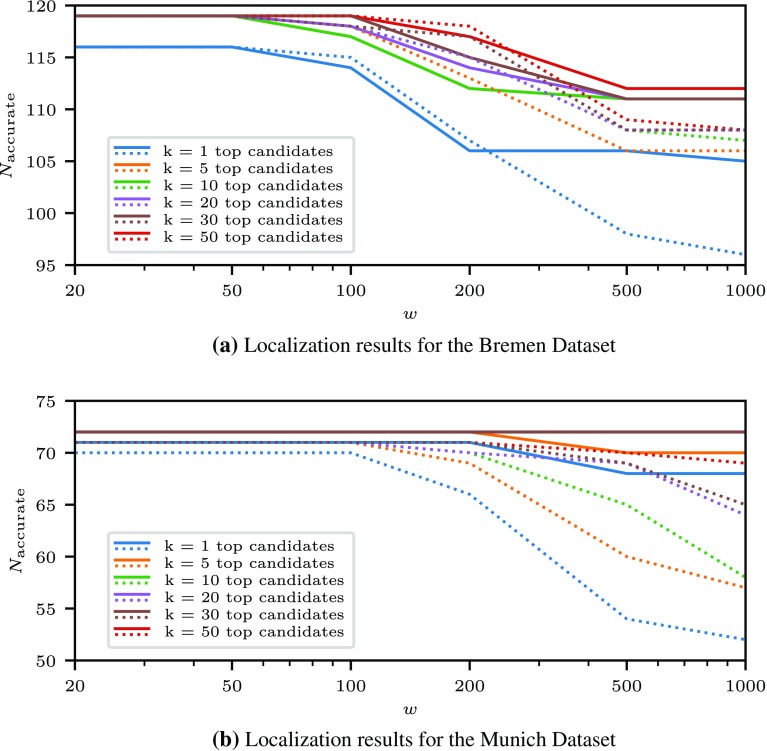
Numbers of accurately localized scenes using two different DCM cost functions for both datasets. Results obtained using the reverse cost function $d_r$ are drawn using solid lines, and those from the standard directional chamfer matching cost function $d_{DCM}$ are drawn dotted. The plot indicates the number of scans $N_{accurate}$ where one among the *k* best-rated candidates within a radius of a given size *w* around the ground truth position is accurate

**Fig. 6:**
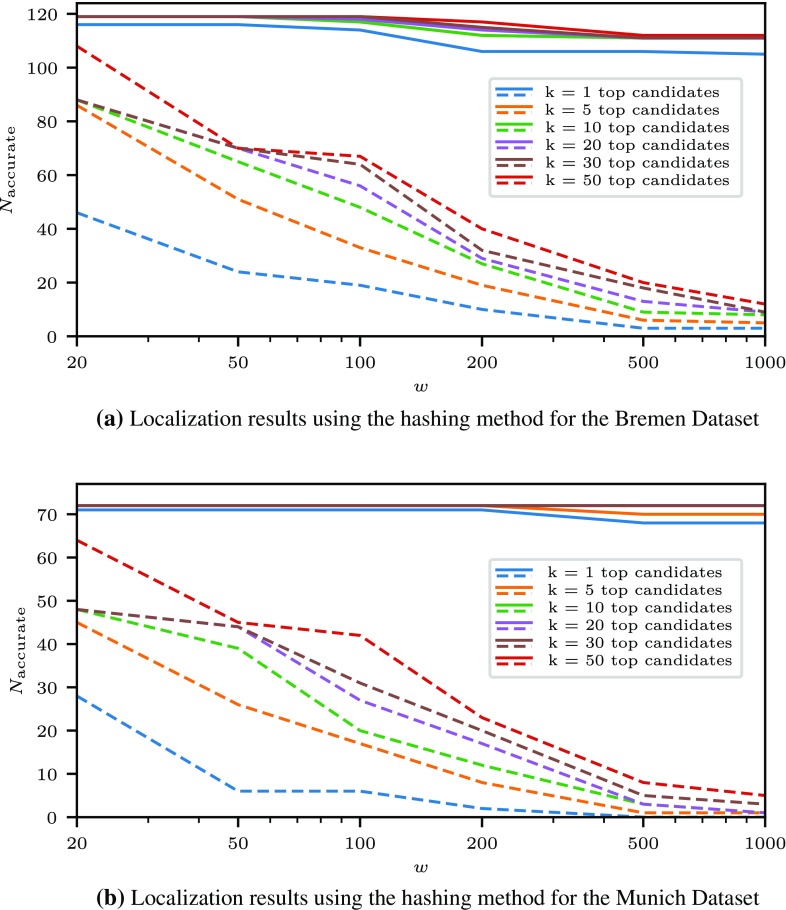
Numbers of accurately localized scenes using the hashing method (dashed lines) and the reverse DCM cost function (solid lines) for both datasets. The plot indicates the number of scans $N_{\mathrm {accurate}}$ where one among the *k* best-rated candidates within a radius of a given size *w* around the ground truth position is accurate

This evaluation of the results of the proposed localization methods is visualized in Fig. 5. It compares the number of accurate best-ranked scans, depending on the size of the search area, for the standard cost function $d_{DCM}$, and the newly proposed reverse cost function $d_r$. The results for the other two newly defined cost functions $d_f$ and $d_s$ were slightly worse than with the reverse cost function, but still outperformed the standard DCM cost $d_{DCM}$, so the individual results are omitted for brevity. Fig. 6 presents a comparison of the hashing-based baseline method and the DCM method using the reverse cost function using the same analysis method.

**Fig. 7:**
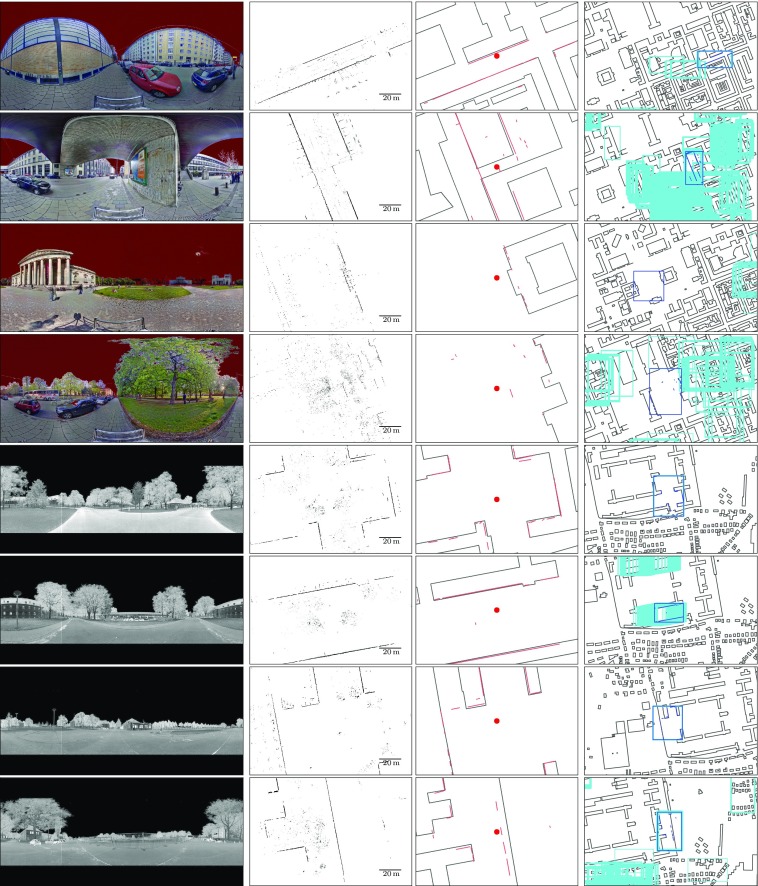
Example results of the line extraction and localization. Images from left to right: Color/intensity image; projection of building points to the ground plane; extracted building segments and OpenStreetMap building map at ground truth pose (indicated by the red dot); section of the localization result: area covered by the observations of the ground truth pose framed in dark blue, candidates for alternate poses in light blue. The top four rows of images show scans from the Munich dataset; the lower four from the Bremen dataset. For a detailed description of the example cases, please refer to the explanation in Sect.  4.3

Figure 7 gives an overview over the variability of scans contained in the dataset and an illustration of the nature of the results of the localization procedure. The first two rows show scans from the Munich data set where the localization provides accurate candidates, while the scans in the second two rows cover wide open areas that do not provide a sufficient number of salient features to allow the retrieval of accurate pose candidates, so the localization fails in these two cases. The fifth, sixth and seventh row show examples of successful localization from the Bremen dataset. The bottom row shows an example of a discrepancy between the observed reality and the map, since a temporary building site fence has been set up at a distance from the corresponding structure in the map. Nevertheless, candidate poses are also generated in the vicinity of the correct localization result.

The pipeline of operations is run on a largely non-optimized python implementation wrapping a modified version of the FDMC implementation of Liu et al. (2010) for matching and cost computations. A cursory analysis of the computational properties of the processing was carried out in single-threaded computation on a Intel Quadcore i5 CPU at 3.3GHz with 16GB RAM. In this analysis, it can be expected that each operation can be sped up considerably with careful optimization. With the current implementation, the computation of the histogram of oriented occupied cells and the line extraction take on average 54 and 220ms, respectively. Building the distance transform tensor for the first FDCM step, which needs to be done once per dataset, takes 35 and 155 s for the maps covering the Munich and Bremen datasets, respectively. This computation time depends largely on the size of the map and the number of elements it contains, as well as the grid size chosen for the candidate extraction. Matching the observed lines to the full building outline map takes 60 s on average. For the candidate selection process, filtering all candidates takes 34 ms per scan. Further computation times are given per candidate that is evaluated; hence, computation times for the candidate selection process can be adapted by limiting the number of candidates that are being evaluated with further cost computation. Computation times for retrieving the visible lines for a candidate pose is 210 ms on average, and computing the reverse costs takes 620 ms per scan to compute the distance transform tensor, and less than 1 ms to compute the cost per candidate. Computing the forward cost takes on average 460 ms per candidate. The reason for this is that the distance transform tensor needs to be computed for each evaluation since the template for the matching changes with each candidate. In addition to the room for computational optimization, it can also be noted that the candidate evaluation can very easily be computed in parallel.

### Discussion of results

As it can be seen by inspecting the results, the proposed method generates an accurate highest-ranking pose candidate even for large search areas in a majority of cases. Taking into account a larger number of high-ranked candidates improves upon this result, which can be useful in applications such as generating an initial distribution of pose estimates for the use in a Monte Carlo localization system. The cost functions taking into account the expected observations of the robot consistently improve the result with respect to the DCM template matching method, although at the cost of increased computational cost. In particular, as it can be seen in Fig. 5, the reverse cost function produces more stable localization results as the search area increases in comparison to the standard DCM cost function. As displayed in Fig.  6, the proposed method also outperforms the simpler approach based on geometric hashing, which is nevertheless able to localize laser scans accurately within smaller areas. This is useful if a position estimate for example from GPS is available, but does not provide satisfactory results for larger areas.

As illustrated in Fig. 7, two causes for failure of the method are the lack of reliable features in areas that contain few buildings, and mismatches between the map and the environment caused by errors in the map or temporary changes in the environment. Results also show that the proposed method works successfully even in instances where no building corners are visible, for example in cases where only sides of a large buildings, the corners of which are outside the sensor range, are visible, or in situations where building corners are occluded. This is not possible with methods relying on geometric hashing or similar techniques, which rely on accurate building corner locations.

## Conclusion

In this article, we have shown an approach to estimate the pose of a robot in a global coordinate frame based on only a laser scan and a map containing building outlines and street network data. The evaluation has shown that this approach performs well on a large part of the data used for experimentation, which includes urban scenes with varying characteristics. It has been demonstrated that explicitly comparing the expected observation with the actual sensor data by including visibility analysis in the cost function benefits the localization accuracy.

The presented approach could be improved in a number of ways. Freely accessible databases offer much more semantically annotated data than what is used in this approach. For example, detecting the area covered by roads and paths could be used as an additional feature. Building heights, which are annotated in some maps, could be used to make the candidate selection process more concise and to generate a more accurate representation of the expected observation.

The presented system can also serve as the basis for other robotic applications, and be used in connection with other sensors. For example, the generated pose candidates with their associated costs can be used to provide the initial pose distribution for a SLAM pipeline. While the matching method has been described and tested on scale-invariant laser data, it can also be applied to building outlines extracted from camera images using computer vision methods, when scale is added as an additional degree of freedom to the first DCM search step.

## Footnotes

1 by Prashant K.C., Dorit Borrmann, Jan Elseberg, and Andreas Nüchter, retrieved from the *robotic 3D scan repository* http://kos.informatik.uni-osnabrueck.de/3Dscans/.
